# Supplementary material for: Strong and ductile high temperature soft magnets through Widmanstätten precipitates
Source: Nat Commun. 2023 Dec 9;14:8176. doi: 10.1038/s41467-023-43953-1 (PMC10710466; doi:10.1038/s41467-023-43953-1)
Supplement: Supplementary file 1 — Supplementary Information file [file 41467_2023_43953_MOESM1_ESM.pdf]

Supplementary Materials for

Strong and ductile high temperature soft magnets through Widmanstätten precipitates

Liuliu Han[1], Fernando Maccari[2], Ivan Soldatov[3], Nicolas J. Peter[1], Isnaldi R. Souza Filho[1], Rudolf Schäfer[3], Oliver Gutfleisch[2], Zhiming Li[4], Dierk Raabe[1]

1. Max-Planck-Institut für Eisenforschung, Max-Planck-Straße 1, 40237 Düsseldorf, Germany
2. Department of Material Science, Technical University of Darmstadt, 64287 Darmstadt, Germany
3. IFW Dresden, Institute for Metallic Materials, Helmholtzstr. 20, 01069 Dresden, Germany
4. School of Materials Science and Engineering, Central South University, 410083 Changsha, China

Correspondence to l.han@mpie.de (L.H.), lizhiming@csu.edu.cn (Z.L.), d.raabe@mpie.de (D.R.)

The file includes:

Supplementary Text
Figs. S1 to S15
Supplementary References

Supplementary Text

Evaluation of strengthening mechanisms

Our alloy design strategy of introducing micrometer-sized precipitate arrays of Widmanstätten type in an fcc matrix enables a considerable improvement in yield strength by 20% (WP-MCA, 640 MPa) compared to that of the precipitate-free reference alloy (HM-MCA, 501 MPa). The improvements are considered to be derived from multiple strengthening mechanisms. Based on our analysis, the contributions of lattice friction stress plus solid solution strengthening (σ_{base}), precipitation strengthening (σ_{ppt}), grain boundary strengthening (σ_{gb}) and dislocation hardening (σ_{d}) in the WP-MCA are quantitatively estimated to be 461.7 MPa, 146.5 MPa, 30.3 MPa and 5.2 MPa, respectively. Generally, two mechanisms are considered to explain precipitate strengthening by the interaction with dislocations, namely, precipitate shearing and dislocation by-passing (Orowan looping). The shearing mechanism dominates when the precipitates are coherent with the matrix, and the size is sufficiently small (a few nanometers). The Orowan mechanism occurs when the dislocations cannot cut the precipitates, so they must circumvent them by bowing out[1,2]. However, neither mechanism is expected to apply in the current WP-MCA based on the experimental observation that dislocations cannot circumvent the micrometer-sized incoherent Widmanstätten-patterned precipitate arrays during deformation. In the present study, the Widmanstätten-patterned precipitates provide a strong barrier to the movement of adjacent dislocations, as evidenced by dislocation pile-ups at the phase interfaces (Fig. 4d and e), which significantly contributes to the high strength of the material. During the later stages of deformation, the locally high pile-up stresses at the phase interfaces activate slip and moderate deformation of the precipitates, often creating microcracks at their intersections (Fig. 4d$_{\mathrm{III}}$ and e$_{\mathrm{III}}$).

The yield strength values of the current WP-MCA were quantitatively evaluated based on the following analytical model[3]:

$$\sigma_{\mathrm{y}} = \sigma_{\mathrm{base}} + \sigma_{\mathrm{gb}} + \sigma_{\mathrm{d}} + \sigma_{\mathrm{ppt}} \qquad (1)$$

where σ_{base} is the sum of lattice friction stress and solid solution strengthening, σ_{gb} is the grain boundary strengthening, σ_{d} refers to dislocation hardening and σ_{ppt} results from precipitation strengthening. The values of σ_{base} for WP-MCA and HM-MCA are considered equivalent because their fcc phases' compositions are close, i.e., for WP-MCA, the composition of the fcc phase is $Fe_{39}Co_{31}Ni_{28}Ta_2$ (at.%), while for HM-MCA, the composition is $Fe_{35}Co_{30}Ni_{30}Ta_5$ (at.%). The calculated strength $\sigma_{\mathrm{base\,(HM)}}$ is 461.7 MPa.

The contribution of σ_{gb} can be evaluated as[4,5]:

$$\sigma_{gb} = \frac{k_y}{\sqrt{D_g}} \quad (2)$$

where k_y (288.5 MPa·μm$^{1/2}$, adopted from the FeCoNi system[6]) represents the Hall-Petch constant, and D_g is the average grain size. Accordingly, the σ_{gb} in the WP-MCA is calculated as 30.3 MPa.

The σ_d caused by the impediment of dislocation motion at the phase boundaries can be described by the classical Taylor model[7]:

$$\sigma_d = \alpha G b \sqrt{\rho} \quad (3)$$

where $\alpha = 0.2$ for fcc structure[8], G is the shear modulus ($G = 84$ GPa, adopted from FeCoNiCr[9]), b stands for the magnitude of the Burgers vector ($b = \frac{\sqrt{2}}{2} \cdot a_l = 0.254$ nm, where a_l is the lattice parameter of the fcc phase acquired from the XRD analysis), and ρ is the dislocation density measured by averaging over 10 ECC images under two-beam conditions as $(2.94\pm0.71)\times10^{12}$ m^{-2} (fig. S8)[10,11]. Accordingly, the σ_d value of the WP-MCA is evaluated to be 5.2 MPa. Based on this analysis, the contribution of σ_{ppt} in the WP-MCA can be approximately estimated by $\sigma_{ppt} = \Delta\sigma_y - \Delta\sigma_{gb} - \Delta\sigma_d$, and a final value of 146.5 MPa is obtained.

Evaluation of the magnetic performance

The saturation magnetization and coercivity of the WP-MCA (115.3 Am^2/kg and 7.9 Oe, respectively) are comparable to those of the HM-MCA (114.2 Am^2/kg and 8.2 Oe, respectively) after introducing 7% (14.5±1.8 vol.%, determined by averaging over 10 ECC images) of micrometer-sized incoherent Widmanstätten-arranged precipitates into the fcc matrix. The mechanisms responsible for the good soft-magnetic properties are unravelled as follows.

We first discuss the maintained saturation magnetization (M_s) in the WP-MCA.

The M_s of the $D0_{19}$ precipitates $M_s(D0_{19})$ were then estimated as follows:

$$93\%\ M_s(\text{fcc}) + 7\%\ M_s(D0_{19}) = 115.3\ \text{Am}^2/\text{kg} \quad (4)$$

The relatively high $M_s(\text{fcc})$ value is mainly derived from the high concentration of ferromagnetic elements, i.e., (Fe+Co+Ni)=98 at.%. The measured bulk M_s for the nominal fcc phase (at 300 K) is 120.5 Am^2/kg, while the M_s of the $D0_{19}$ precipitates calculated is 46.2 Am^2/kg.

The empirical formula based on the theory of spin waves is used to quantify the saturation magnetization (M_s) at elevated temperatures:

$$M_s(T)/M_s(0) = B_J(a) \quad (5)$$

where $M_s(T)$ is the M_s at various temperatures (T=5, 300, 573, 673, 773, 873 K), $M_s(0)$ is the

M_s at absolute zero temperature (0 K). The value of $M_s(0)$ is approximately estimated by measuring the M_s at 5 K ($M_s(0) \approx M_s(5)$). $B_J(a)$ is the Brillouin formula described as:

$$B_J(a) = \left(1 + \frac{1}{2J}\right) \cdot \coth\left(1 + \frac{1}{2J}\right) \cdot a - \frac{1}{2J} \cdot \coth\left(\frac{a}{2J}\right) \quad (6)$$

where J represents the total angular momentum quantum number of the atom and a is defined as:

$$\frac{M_s(T)}{M_0} = \tanh\left[\frac{M_s(T)}{M_0}\left(\frac{\theta_C}{T}\right)\right], (J = \frac{1}{2}) \quad (7)$$

$$\frac{M_s(T)}{M_0} = \coth\left[3\frac{M_s(T)}{M_0}\left(\frac{\theta_C}{T}\right)\right] - \frac{1}{3}\frac{M_0}{M_s(T)}\left(\frac{T}{\theta_C}\right), (J = \infty) \quad (8)$$

$$\frac{M_s(T)}{M_0} = \frac{3}{2}\coth\left[\frac{9M_s(T)}{4M_0}\left(\frac{\theta_C}{T}\right)\right] - \frac{1}{2}\coth\left[\frac{9M_s(T)}{4M_0}\left(\frac{\theta_C}{T}\right)\right], (J = 1) \quad (9)$$

where $M_s(T)/M_s(0)$ is evaluated by fitting different J values as a function of the reduced temperature (T/T_c), T_c is the Curie temperature.

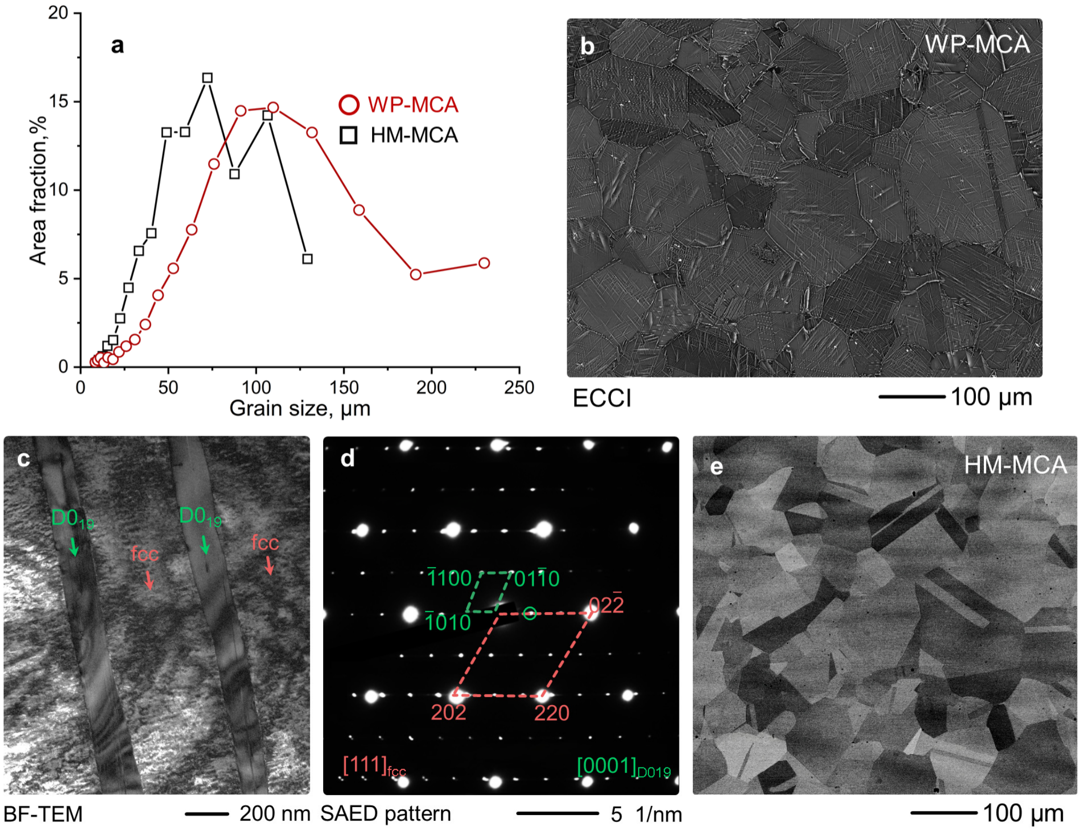

Fig. S1.

EBSD analysis for the undeformed WP-MCA. a Grain size distribution acquired by EBSD analysis. For comparison, the distribution of the HM-MCA is also included. **b** Low magnification ECC imaging analysis. **c** Bright-field TEM analysis of the WP-MCA. The sample region is identical to that denoted by the yellow frame in Fig. 1d. **d** The corresponding SAED pattern. **e** Low magnification ECCI analysis showing the single-phase HM-MCA.

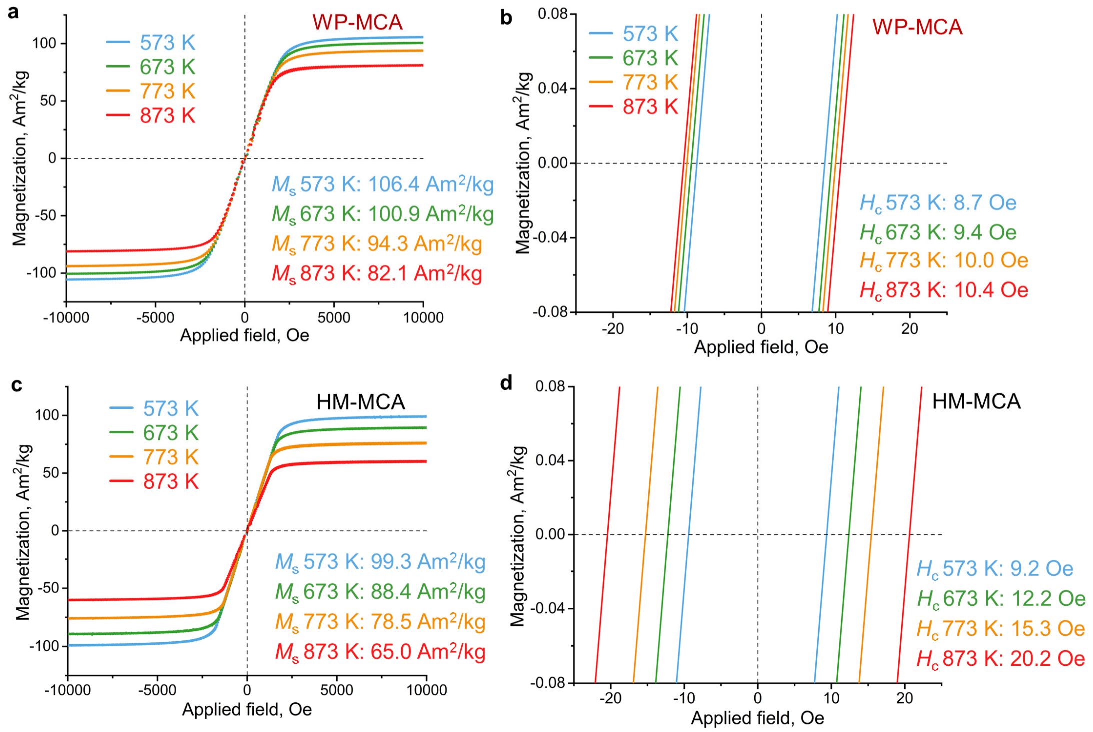

Fig. S2.

High-temperature magnetic response of the current MCAs. Hysteresis loops acquired up to ±10000 Oe of **a** WP-MCA and **b** Corresponding enlarged view showing the intrinsic coercivity at various temperatures from 573 K to 873 K. **c** HM-MCA and **d** Corresponding enlarged view showing the intrinsic coercivity at various temperatures from 573 K to 873 K.

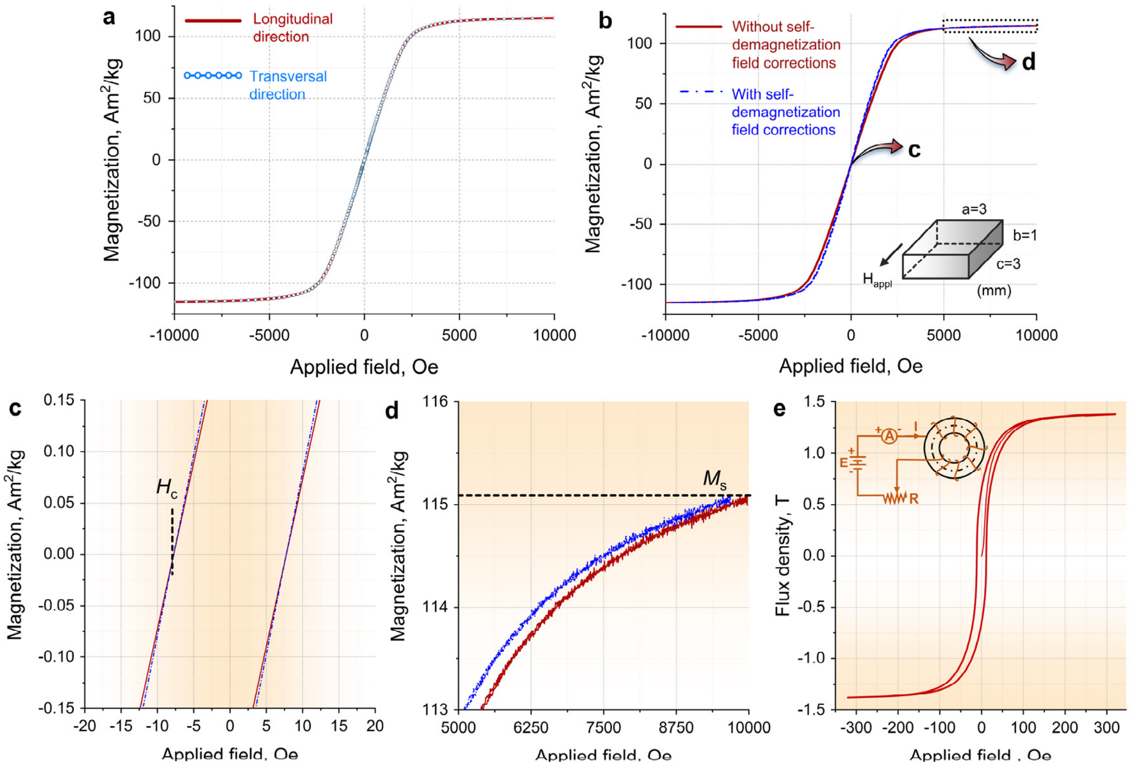

Fig. S3. Crystallographic and demagnetization effect on magnetic performance.

a Hysteresis loop of the WP-MCA acquired along the longitudinal and transversal directions up to ±10000 Oe at room temperature. **b** Measured and corrected hysteresis loops of the WP-MCA along the longitudinal direction acquired up to ±10000 Oe at room temperature. The right-bottom inset shows the schematic illustration of the specimen dimension for calculating self-demagnetization correction factors along the applied magnetic field direction in the PPMS. The enlarged views showing the values of **c** Coercivity and **d** Saturation magnetization are not affected by the shape of the hysteresis loop in the current work. **e** Closed-circuit measurements showing the magnetic flux density in response to the applied field. The left-top inset shows the schematic illustration of the specimen winding and experiment set-up.

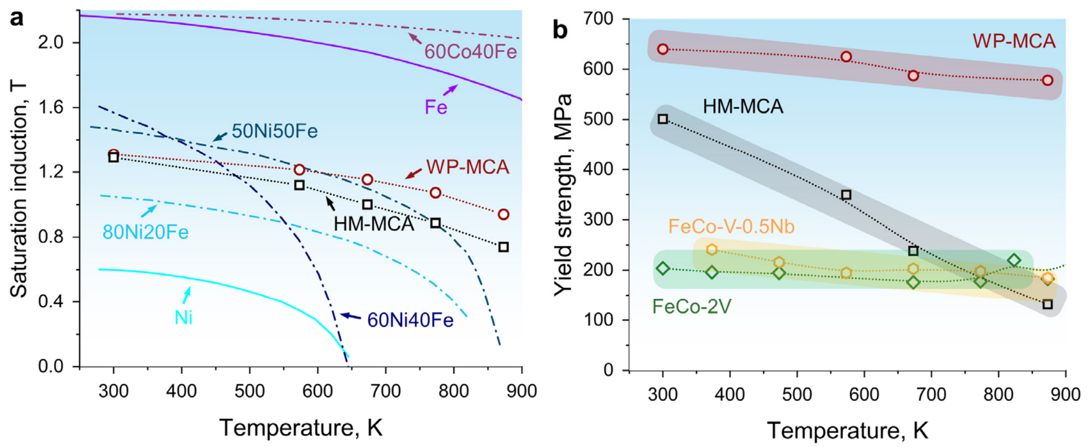

Fig. S4. Plots comparing the temperature dependence of the a Saturation induction and b Yield strength of the WP-MCA with several typical commercial high-temperature SMMs. The temperature dependence of saturation induction (B_s) of the conventional magnetic Ni, 80Ni20Fe, 60Ni40Fe, 50Ni50Fe, Fe and 60Co40Fe materials[12] are compared. The temperature dependence of yield strength of the FeCo-2V[13] and FeCo-V-0.5Nb[14] are compared.

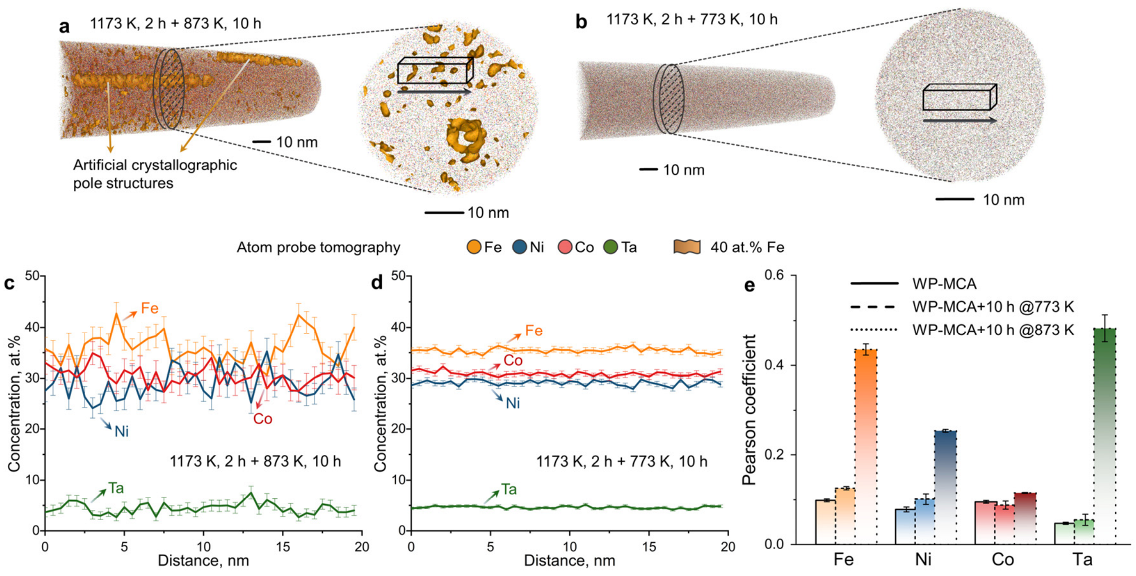

Fig. S5.

APT analysis of the MCAs after further exposure at high temperatures. a Reconstructed APT map showing the elemental distributions after further annealing the WP-MCA at 873 K for 10 h. The corresponding plane view of a 2.5 nm-thick slice from the tip is shown. The interface is highlighted with 40 at.% Fe. It should be noted that the needle-like isosurface is the depleted artificial region, indicating typical crystallographic pole structures[15]. **b** Reconstructed APT map of the MCA after annealing the WP-MCA at 773 K for 10 h and the corresponding plane view of a 2.5 nm-thick slice from the tip. The concentration of Fe is lower than 40 at.% in the whole tip. **c** and **d** 1D compositional profiles from the rectangular region of interest along the black arrows in **a** and **b**, respectively. **e** Average normalized homogenization parameter analysis. The error bars are estimated as described in Methods.

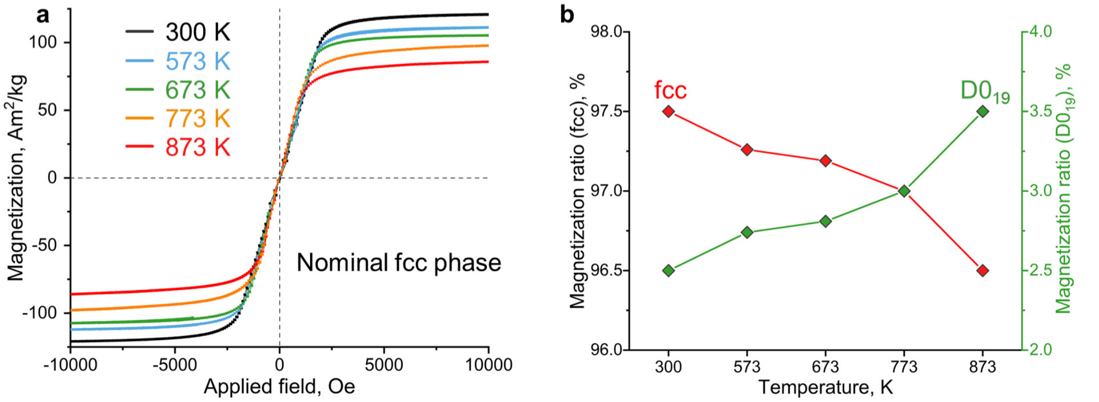

Fig. S6.

Room- and high-temperature magnetic response of the nominal fcc matrix phase ($Fe_{39}Co_{31}Ni_{28}Ta_2$, at.%) in the WP-MCA using an as-cast ingot (50 g). a Hysteresis loops measured at different temperatures (300 K, 573 K, 673 K, 773 K and 873 K). **b** Magnetization contributions of the fcc and $D0_{19}$ phases to the overall saturation magnetization of the WP-MCA at different temperatures.

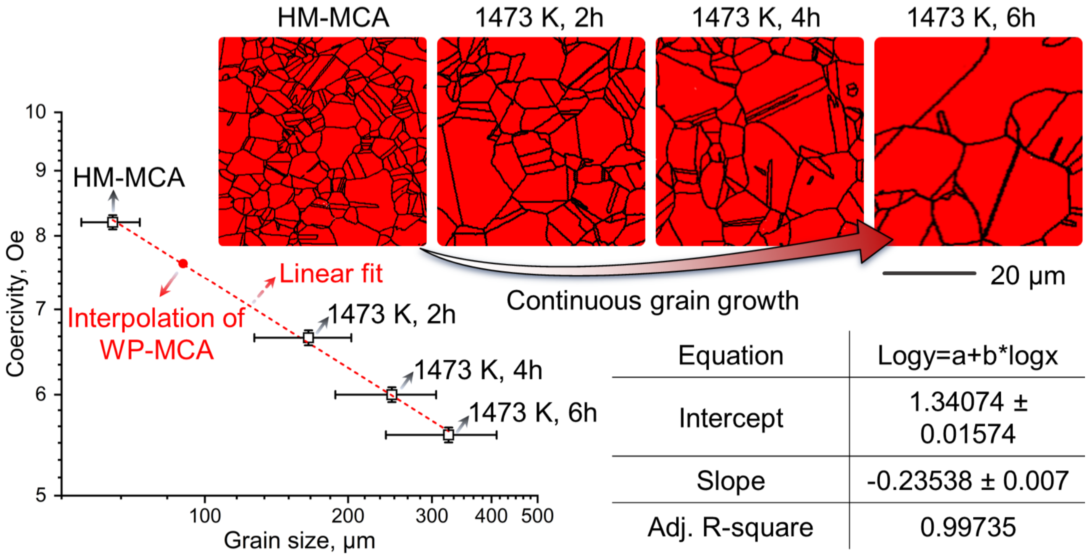

Fig. S7.

Interpolation of the reciprocal of grain size on coercivity only considering the grain size effect on the coercivity. Linear fit of grain size and coercivity of the $Fe_{35}Co_{30}Ni_{30}Ta_{5}$ (at.%) multicomponent material with different annealing times at 1473 K. The inserted EBSD phase maps illustrate the corresponding evolution of grain structures with increasing annealing time. The error bars are averaged by multiple EBSD measurements.

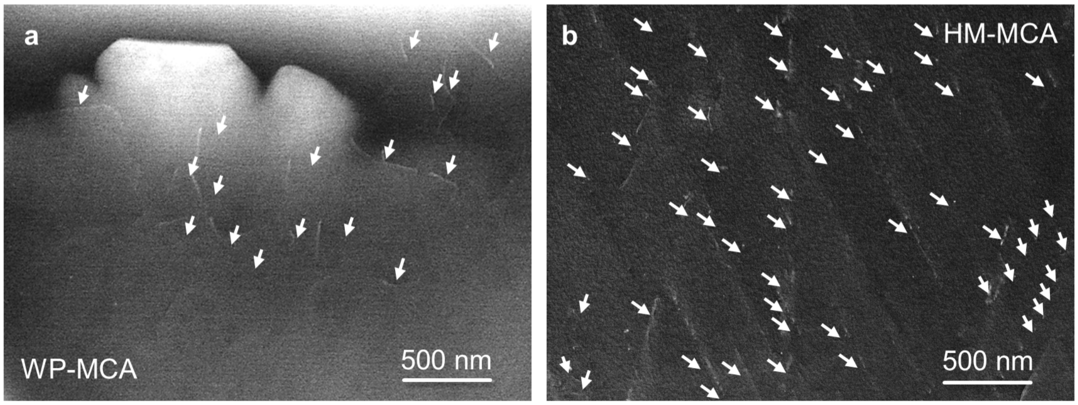

Fig. S8.

Typical ECC images showing the dislocation arrangements in the alloy samples before tensile deformation. The dislocation density of each alloy sample was measured by averaging over 10 ECC images. **a** WP-MCA. **b** HM-MCA.

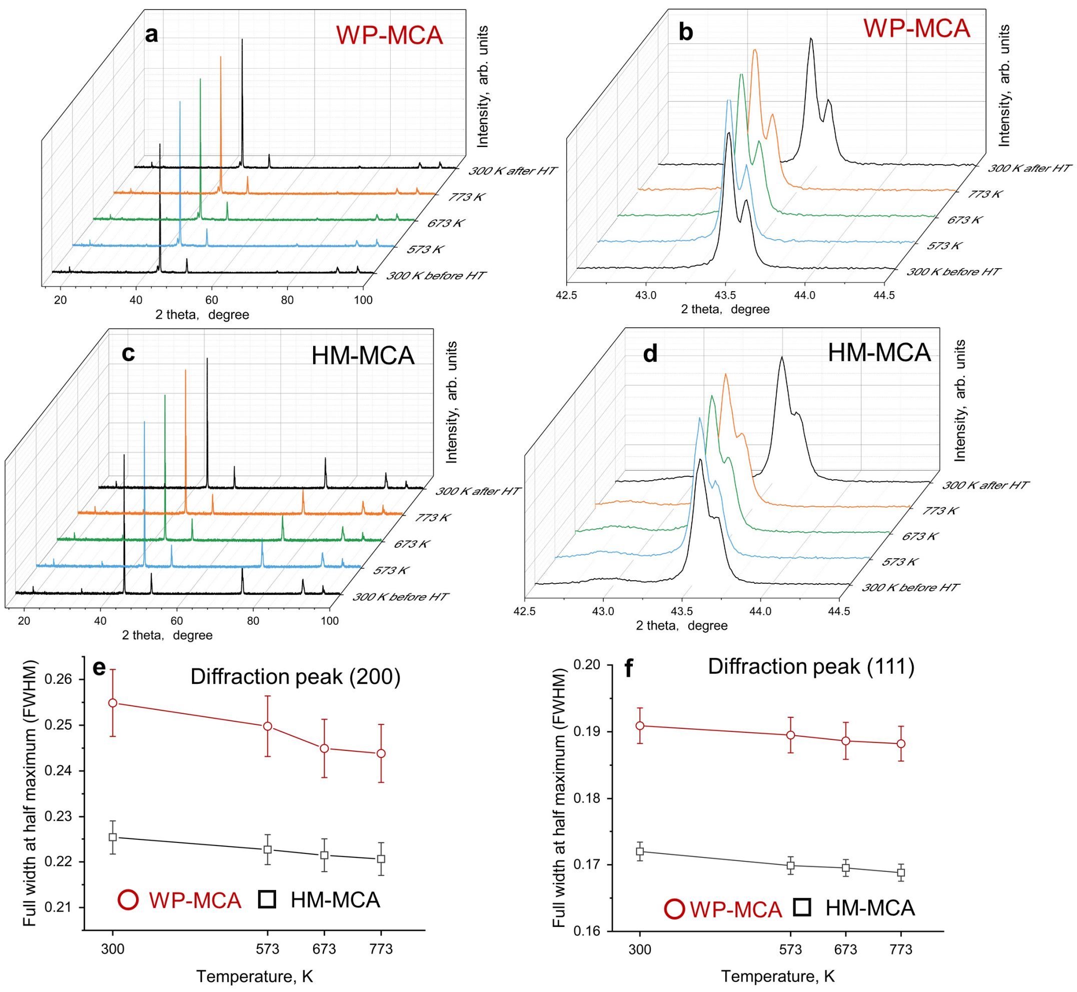

Fig. S9.

High-temperature XRD analysis. The measurements were performed at room temperature and 573 K, 673 K, 773 K, then back to room temperature. **a** WP-MCA and **b** Corresponding enlarged view of the (111) diffraction peak. **c** HM-MCA and **d** Corresponding enlarged view of the (111) diffraction peak. **e** and **f** are the measured full width at half maximum value of the WP-MCA and HM-MCA of (111) and (200) diffraction peaks, respectively. The decrease in full width at the half maximum value of the HM-MCA material from 773 K to 300 K are 2.1% and 1.6% for the (200) and (111) peaks by fitting, respectively. While the values for the WP-MCA material are 4.3 and 1.8%, respectively. The additional reflections near the (111) peak from the hexagonal crystal structure make the fitting less reliable than that for the (200). All standard deviations are determined by Rietveld simulation.

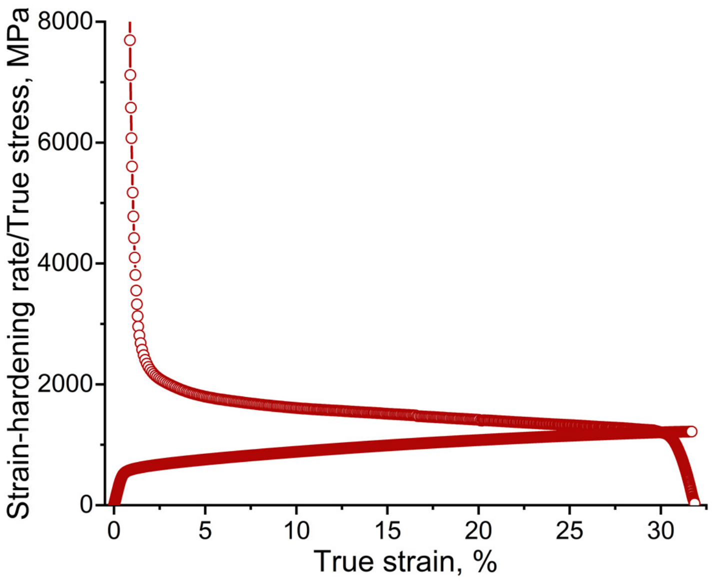

Fig. S10.

Strain-hardening rate and true stress as a function of the true strain of the WP-MCA at room temperature. During plastic straining, a linear increase in true stress and a monotonic two-stage decrease in strain-hardening rate are observed.

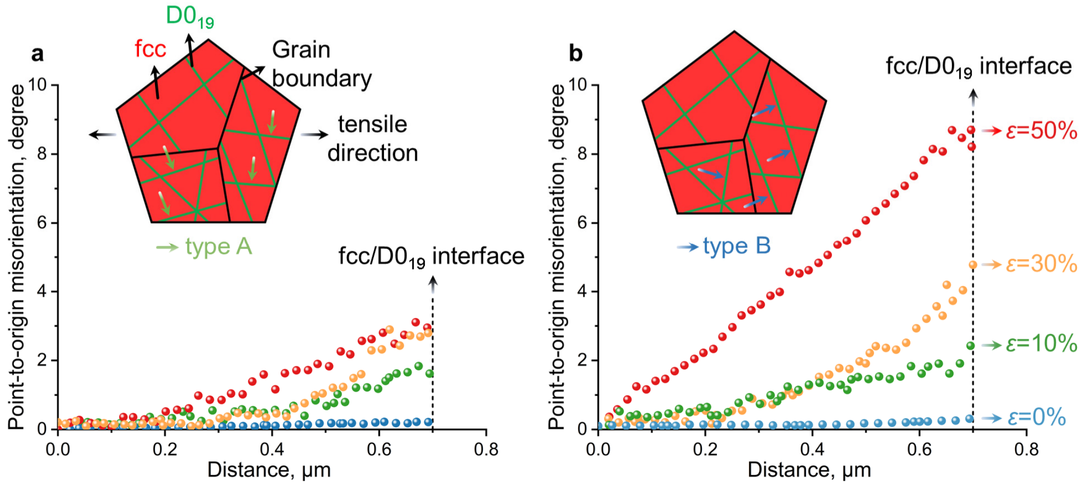

Fig. S11.

Point-to-origin misorientation analysis. Two types of misorientation are analyzed. **a** The average misorientation acquired perpendicular towards the Widmanstätten-pattern precipitates parallel to the tensile direction within 5 degrees (see green arrows in the inset). **b** The average misorientation acquired perpendicular towards the Widmanstätten-pattern precipitates that are verticle to the tensile direction within 5 degrees (see blue arrows in the inset). The undeformed microstructure shows negligible misorientation when perpendicularly approaching the Widmanstätten-pattern precipitates that are verticle to the tensile direction (within 5 degrees), compared to the deformed state—the analysis confirming that the observed build-up of local misorientations is mainly derived from the dislocation activities.

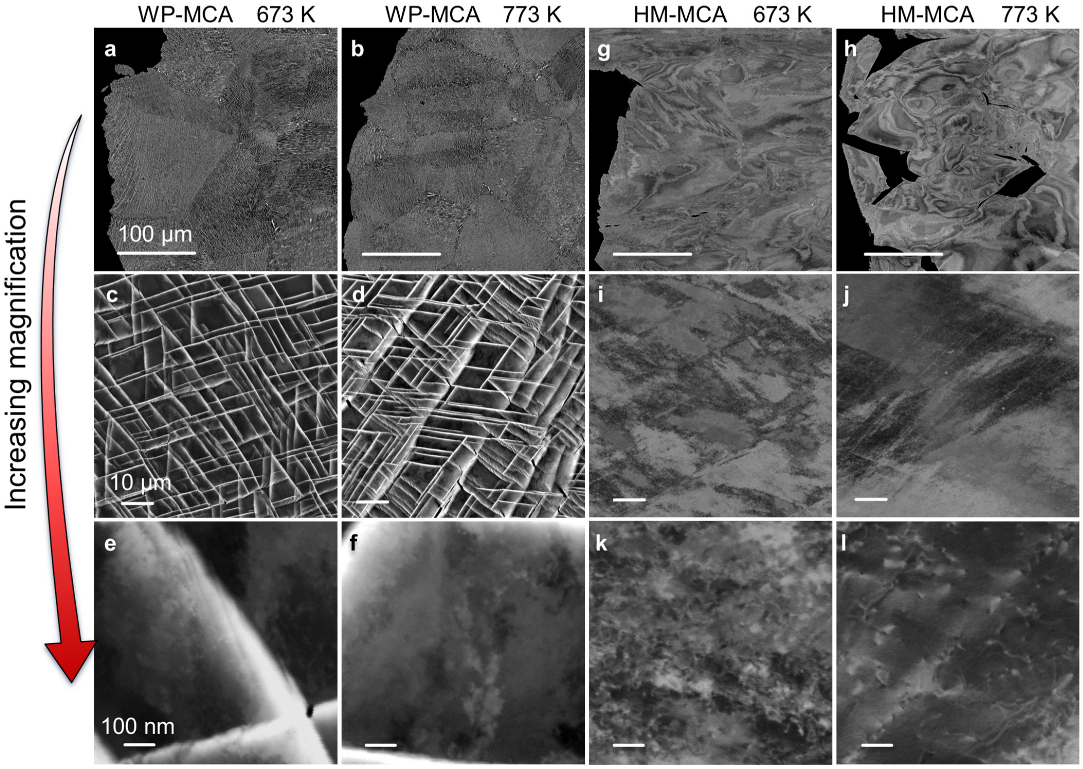

Fig. S12.

ECC imaging analysis showing the deformation of microstructure after high-temperature tensile measurements. **a**, **c**, **e** The cross-sectional fracture of the WP-MCA at 673 K from low to high magnifications. **b**, **d**, **f**, and **g**, **i**, **k**, and **h**, **j**, **l** are the corresponding images of the WP-MCA measured at 773 K, HM-MCA measured at 673 K and HM-MCA measured at 773 K, respectively.

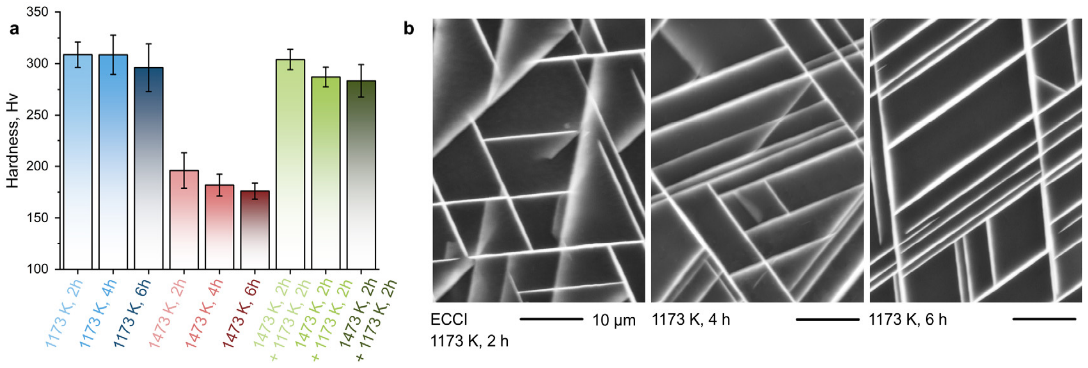

Fig. S13.

Effect of different isothermal treatment processing on the hardness and microstructure of the studied alloy. a Hardness response of the WP-MCA including (i) alloy variants with different annealing times (1473 K for 10 min followed by 1173 K for 2 h, 4 h and 6 h); (ii) alloy variants with different homogenization times (1473 K for 2 h, 4 h and 6 h); (iii) alloy variants with different homogenization times (1473 K for 2 h, 4 h and 6 h) then followed by the same annealing processing as the current WP-MCA (1173 K for 2 h). The standard deviation of the hardness value is averaged using at least ten indents. **b** Microstructural evolution of alloy variants after different annealing times at 1173 K. Prolonging the annealing time to 6 h has no obvious effect on the precipitation morphology. This indicates the excellent thermal stability of the $D0_{19}$ precipitate.

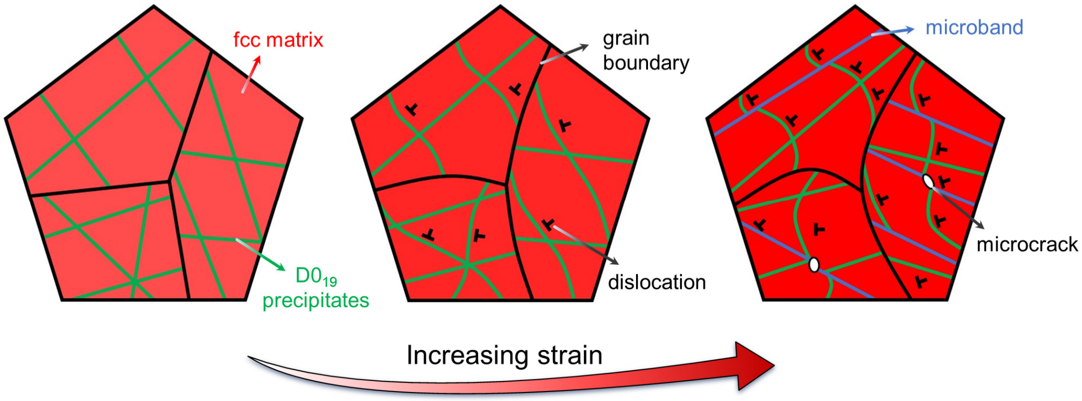

Fig. S14.

Schematic illustration of the micro-processes during different plastic and hardening stages. The generation and pile-ups of dislocations in the early deformation stage lead to stress concentrations at the D0$_{19}$/fcc interface, leading to the deformation of the D0$_{19}$ precipitates. Further straining favours the activation of relatively sharp microbands. As a result, the dislocation accumulations at the sheared D0$_{19}$ intersections lead to stress localization and forming pores and micro-cracks.

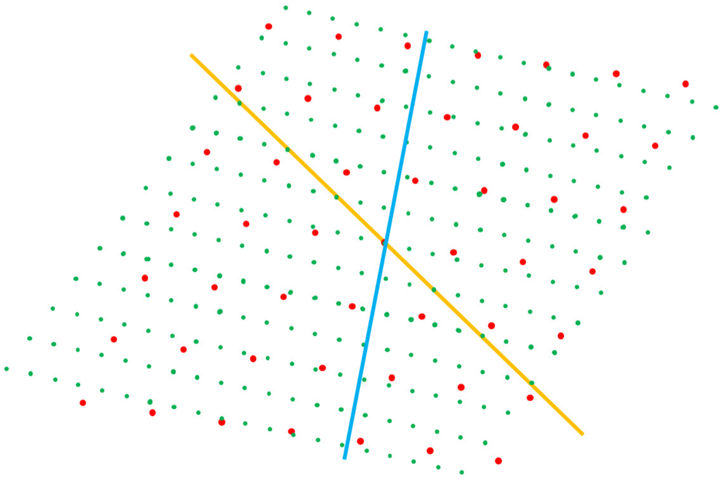

Fig. S15.

Coincidence site lattice analysis. The periodic coincidence sites are taken from the diffraction spots of the fcc matrix (red dots) and $D0_{19}$ precipitates (green dots). The blue and yellow lines are taken from the fcc/$D0_{19}$ interface (Fig. 1f) and sheared interface (Fig. 4h), respectively. No overlapping of these two lattice spots is observed within the periodic sites, indicating high mismatch and low coherency between these two phases.

Supplementary References

1. C. Booth-Morrison, D. C. Dunand, D. N. Seidman, *Acta Mater*. **59**, 7029–7042 (2011). https://doi.org/10.1016/j.actamat.2011.07.057

2. R. C. Reed, The superalloys: fundamentals and applications, *Cambridge*, (2008). https://doi.org/10.1017/CBO9780511541285

3. N. Kamikawa *et al. Acta Mater.* **83**, 383–396 (2015). https://doi.org/10.1016/j.actamat.2014.10.010

4. E. O. Hall, *Proc. Phys. Soc. B* **64**, 747 (1951). https://doi.org/10.1088/0370-1301/64/9/303

5. N. J. Petch, *J. Iron Steel Inst.* **174**, 25–28 (1953).

6. X. L. An *et al. J. Alloys Compd.* **807**, 151698 (2019). https://doi.org/10.1016/j.jallcom.2019.151698

7. G. I. Taylor *Phys. Soc. A* **145**, 362–387 (1934). https://doi.org/10.1098/rspa.1934.0106

8. T. H. Courtney, Mechanical Behavior of Materials. *Waveland* (2005). ISBN: 978-1-57766-425-3

9. Z. Wu, H. Bei, G. M. Pharr, E. P. George, *Acta Mater.* **81**, 428–441 (2014). https://doi.org/10.1016/j.actamat.2014.08.026

10. I. Gutierrez-Urrutia, D. Raabe, *Scr. Mater.* **66**, 343–346 (2012). https://doi.org/10.1016/j.scriptamat.2011.11.027

11. I. Gutierrez-Urrutia, D. Raabe, *Mater. Sci. Forum* **783–786**, 750–754 (2014).https://doi.org/10.4028/www.scientific.net/MSF.783-786.750

12. R. M. Bozorth, Ferromagnetism. (1993). ISBN: 978-0-780-31032-2

13. P. Moine, J. P. Eymery, P. Grosbras, Physica status solidi (b). **46**, 177–185 (1971). https://doi.org/10.1002/pssb.2220460115

14. Ren, L., Basu, S., Yu, R.-H., Xiao, J. Q. & Parvizi-Majidi, A. Mechanical properties of Fe-Co soft magnets. J Mater Sci **36**, 1451–1457 (2001). https://doi.org/10.1023/A:1017588411888

15. B. Gault *et al. Microsc. Microanal.* **28**, 1116–1126 (2022). https://doi.org/10.1017/S1431927621012952
